# A schwannoma of the olfactory nerve: a case report of a diagnostic challenge and review of the literature

**DOI:** 10.1093/jscr/rjag058

**Published:** 2026-02-12

**Authors:** Yehia Nabil, Essam M Youssef, Ahmed Alawamry, Ahmad M Elsharkawy, Hesham R Abdelaziz, Ahmad Alkheder, Mahmoud M Taha

**Affiliations:** Faculty of Medicine, Zagazig University, Zagazig, Egypt; Department of Neurosurgery, Zagazig University, Zagazig, Egypt; Department of Neurosurgery, Zagazig University, Zagazig, Egypt; Department of Neurosurgery, Zagazig University, Zagazig, Egypt; Department of Pathology, Zagazig University, Zagazig, Egypt; Department of Otorhinolaryngology, Al-Mouwasat University Hospital, Damascus University, Damascus, Syria; Faculty of Medicine, Damascus University, Damascus, Syria; Faculty of Medicine, Syrian Private University, Damascus, Syria; Department of Neurosurgery, Zagazig University, Zagazig, Egypt

**Keywords:** olfactory nerve schwannoma, diagnostic challenge, meningioma mimic, skull base tumor, histopathology, immunohistochemistry

## Abstract

Olfactory groove schwannomas are exceptionally rare intracranial tumors that present a significant diagnostic challenge, as they are frequently misidentified as meningiomas on preoperative imaging. We report the case of a 44-year-old woman who presented with a two-month history of headache and blurred vision. Neuroimaging revealed a large, dural-based, homogeneously enhancing extra-axial mass in the left frontal region, strongly suggestive of a meningioma. The patient underwent surgical excision of the lesion. Histopathological examination, however, revealed the classic features of a benign schwannoma, including Antoni A and B areas, nuclear palisading, and Verocay bodies. This diagnosis was confirmed by an immunohistochemical profile that was positive for S100 and negative for Epithelial Membrane Antigen. This case underscores the limitations of radiological diagnosis for skull base lesions and highlights the critical role of histopathological and immunohistochemical analysis in achieving a definitive diagnosis, which is paramount for guiding appropriate management.

## Introduction

Schwannomas are benign, slow-growing tumors arising from peripheral nerve sheath Schwann cells. While common in the head and neck, particularly the vestibular nerve, intracranial olfactory nerve schwannomas are notably rare [1]. Their non-specific presentation—headaches, visual disturbances, and elevated intracranial pressure—overlaps with other skull base pathologies [2]. The olfactory groove is predominantly associated with meningiomas, extra-axial tumors from arachnoid cap cells. Radiologically, both can appear as well-defined, dural-based, enhancing masses on computed tomography (CT) and magnetic resonance imaging (MRI), increasing pre-operative suspicion for meningioma [3]. This mimicry complicates diagnosis, as surgical planning for excision requires accurate pathological identification.

We present a 44-year-old female with an olfactory groove mass, radiologically classified as a meningioma but histopathologically confirmed as a benign schwannoma. This underscores radiological limitations and histopathology’s essential role in definitive skull base tumor diagnosis.

## Case presentation

**Figure 1:**
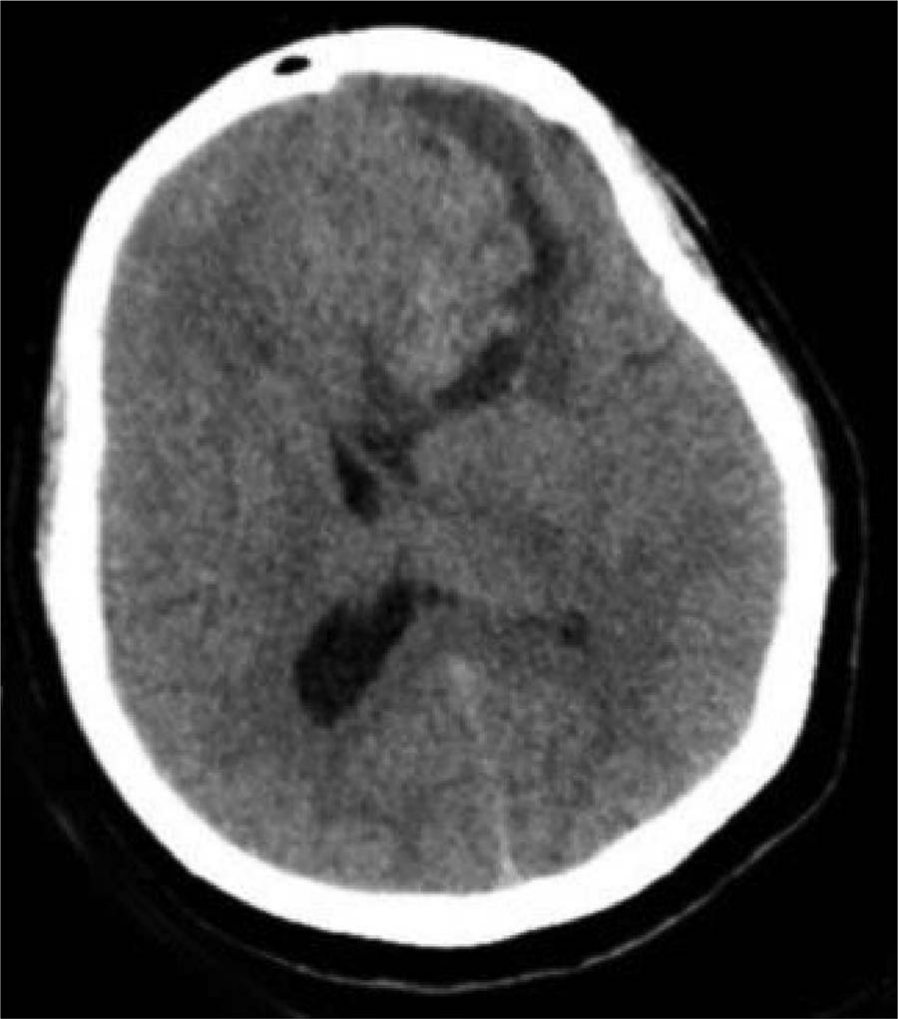
Preoperative CT brain without contrast (axial view) showing a well-circumscribed mass in the right frontal lobe resting on the falx cerebri, causing leftward midline shift with associated vasogenic edema and mass effect.

**Figure 2:**
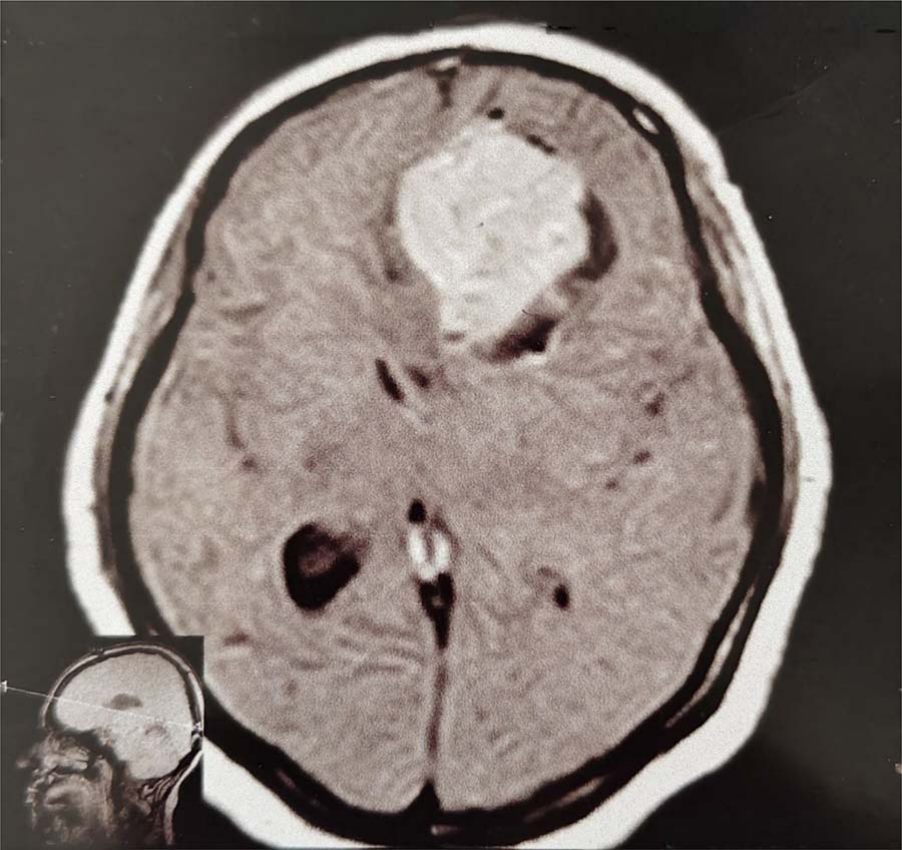
MRI brain without IV contrast (T1 sequence) showing a well-circumscribed hypointense mass with areas of heterogeneity occupying the right frontal lobe, closely related to the midline structures and falx cerebri.

**Figure 3:**
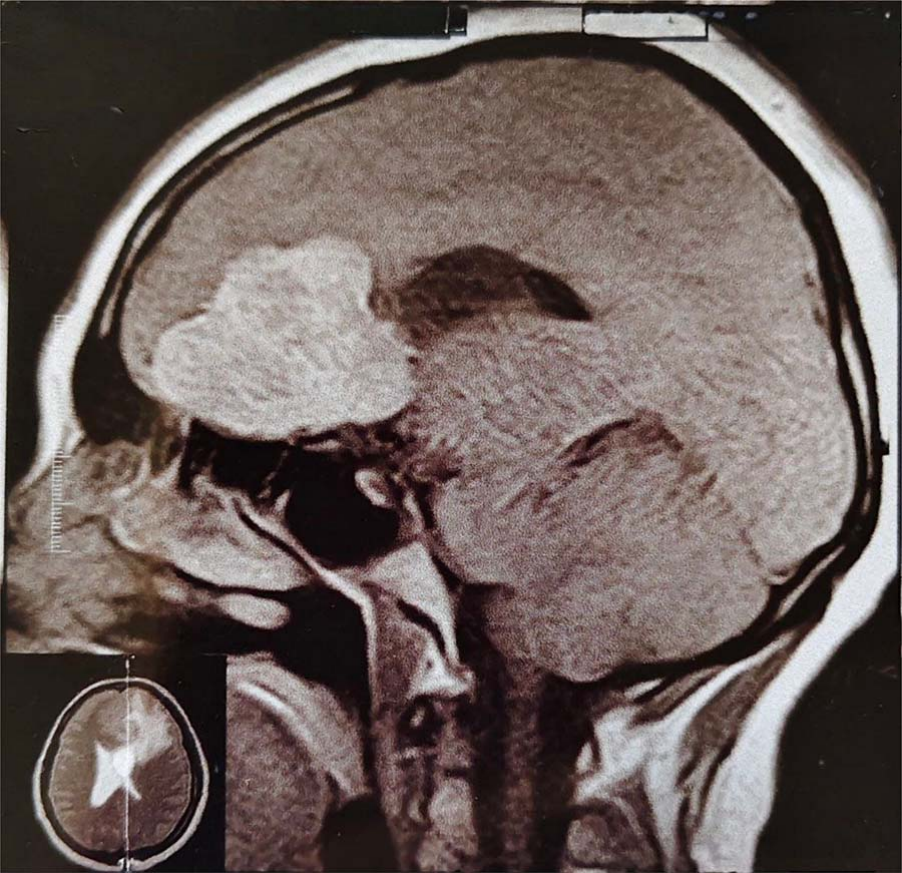
MRI brain with IV contrast (sagittal midline view) showing a homogeneously enhancing mass based on the cribriform plate of the ethmoid, displacing the frontal lobe upward and posteriorly.

**Figure 4:**
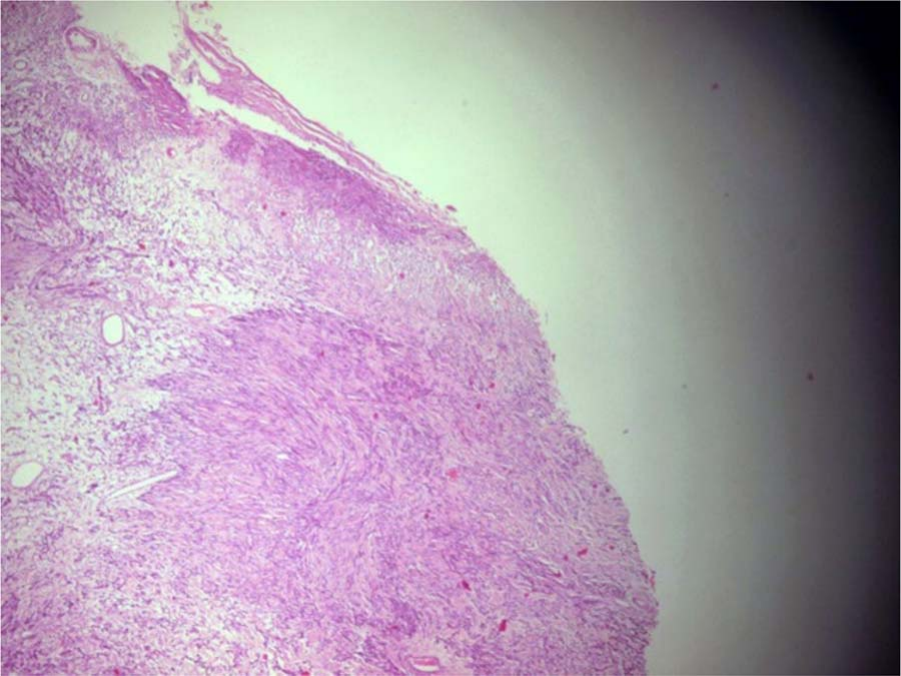
Photomicrograph showing a well-circumscribed, partially encapsulated lesion composed of hypercellular Antoni a areas admixed with myxoid hypocellular Antoni B areas (H&E, ×100).

**Figure 5:**
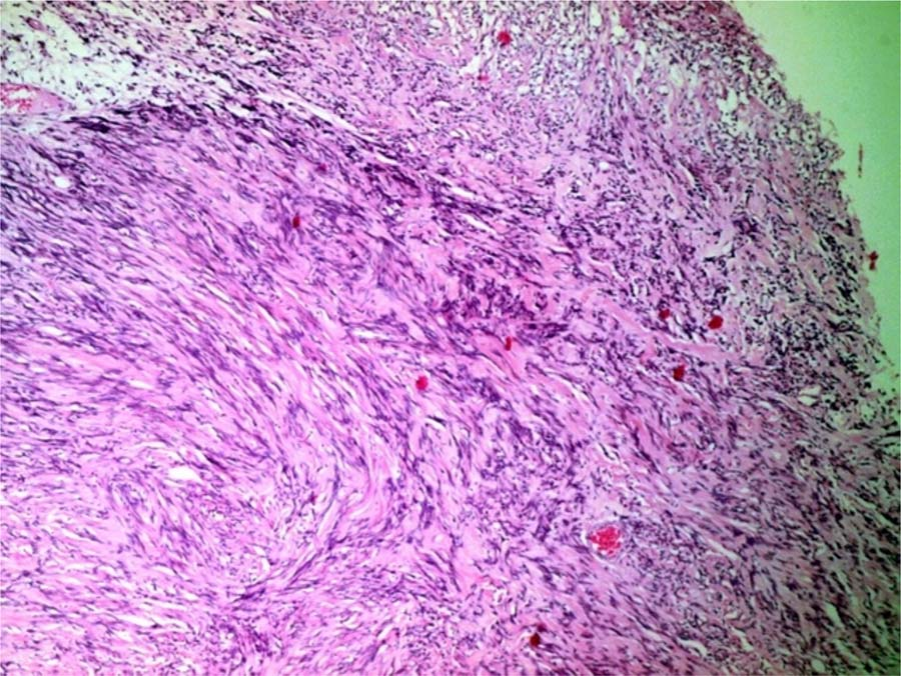
Photomicrograph of hypercellular Antoni a areas showing interlacing bundles of spindle-shaped cells with nuclear palisading (H&E, ×400).

**Figure 6:**
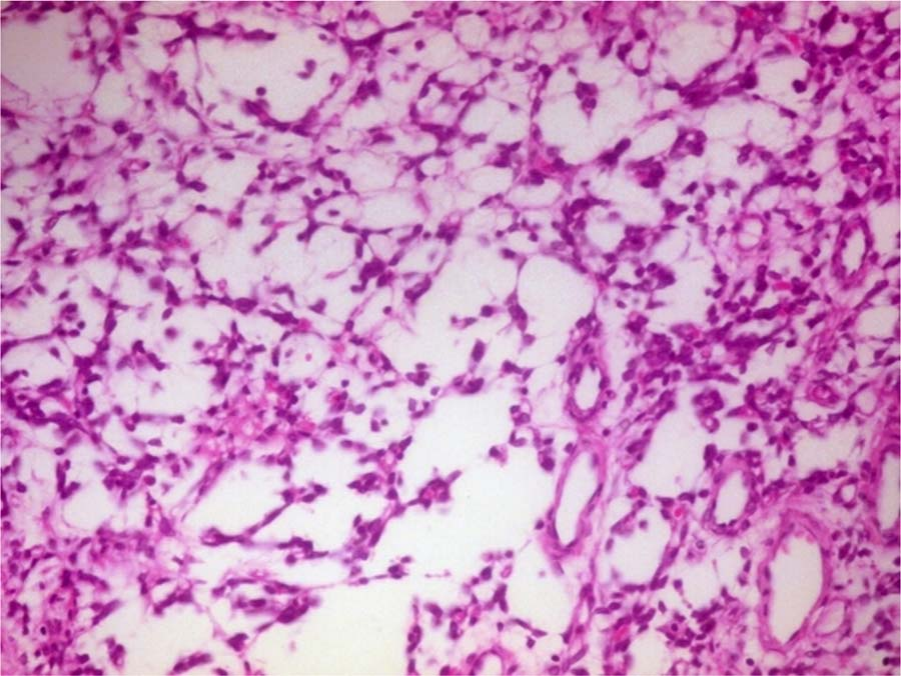
Photomicrograph of hypocellular Antoni B areas showing few cuboidal cells separated by myxoid stroma (H&E, ×400).

**Figure 7:**
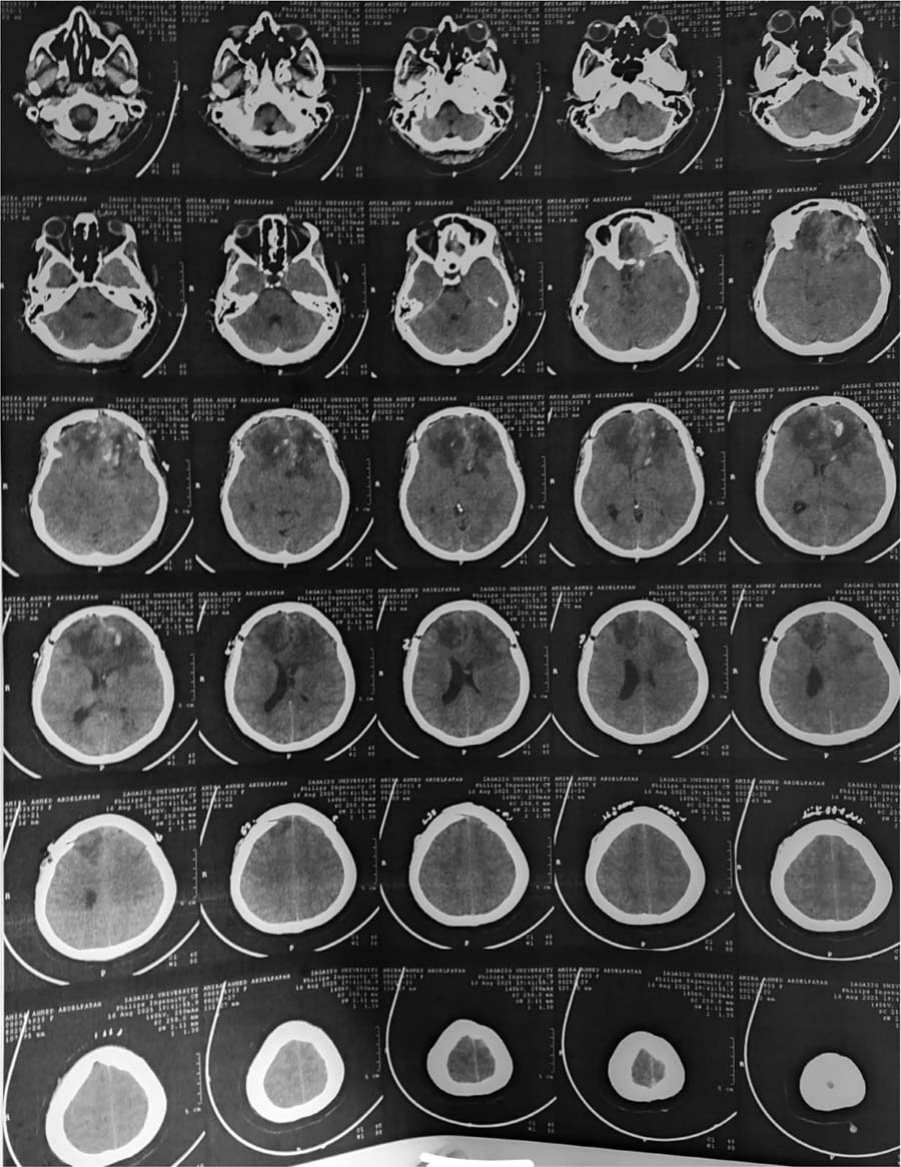
Post-op CT brain showing total excision of the tumor leaving mild post-op oedema at tumor bed.

A 44-year-old right-handed woman presented with a two-month history of headache and blurred vision. Her medical history included hypertension and type 1 diabetes mellitus. She reported gradually worsening intermittent headache and bilateral visual blurring, more pronounced in the right eye. Symptoms exacerbated with physical exertion and were unrelieved by analgesics, leading to her referral. Examination revealed a conscious, alert patient with right eye proptosis. Visual acuity was 6/6 (left eye) and 6/9 (right eye). Fundoscopy identified bilateral Grade 3 papilledema. Non-contrast brain CT demonstrated a well-defined, isodense, dural-based extra-axial lesion in the left frontal region with a cystic component, measuring approximately 5.5 × 4.3 × 4.2 cm. It was associated with mild perilesional edema, mass effect, and a subtle contralateral falx shift. The initial impression was an atypical meningioma (Fig. 1). Subsequent MRI confirmed a large, falcine-based lesion that appeared isointense on T1- (Fig. 2) and T2-weighted images, demonstrating intense homogeneous enhancement post-contrast (Fig. 3). Signal voids suggested calcifications. The patient underwent gross total excision via a bilateral frontal craniotomy. The tumor was intradural and extra-axial, elevating the frontal lobe and was easily accessible; therefore, neither neuronavigation nor other intra-operative localization aids were utilized. Intraoperatively, the olfactory tract could not be clearly identified. A gap in the anterior cranial base was noted, but as there was no evidence of dural invasion or cerebrospinal fluid leak, no sealant was required. Gross examination of the resected specimen revealed a well-circumscribed, white nodule. Microscopically, sections showed benign spindle cell proliferation with alternating Antoni A and Antoni B areas (Fig. 4). Antoni A regions displayed nuclear palisading and Verocay bodies (Fig. 5), while Antoni B areas were edematous and myxoid (Fig. 6). No mitotic figures or atypia were seen. The definitive diagnosis was a benign olfactory groove schwannoma, notable given the initial radiological suspicion of meningioma. Her postoperative course was uneventful. A postoperative non-contrast CT brain obtained 48 hours after surgery confirmed gross total resection (Fig. 7). She was discharged with scheduled follow-up to monitor recovery and visual symptoms.

## Discussion

Olfactory groove schwannomas (OGS) are rare and diagnostically challenging, as the olfactory nerve contains few Schwann cells, the typical origin of schwannomas. A 44-year-old woman had a large left frontal extra-axial lesion, initially diagnosed radiologically as a meningioma but later confirmed histopathologically as a benign schwannoma, illustrating this diagnostic dilemma and aligning with literature trends [4–7].

The initial radiological diagnosis of meningioma was understandable. The lesion demonstrated features of an atypical meningioma on CT and MRI: a well-defined, dural-based, extra-axial mass with significant homogeneous contrast enhancement, edema, and mass effect [8]. This diagnostic error is common; most OGS cases are misdiagnosed preoperatively as olfactory groove meningiomas [4–7, 9–12]. Shared radiological features include a dural base, isointensity on T1/T2 sequences, and homogeneous enhancement [4, 8, 9]. However, certain subtle indicators can suggest schwannoma. Cystic components, as in our case and others [4, 8], and the absence of hyperostosis or a prominent dural tail [10, 11], favor OGS. Bone erosion, noted by Shenoy *et al*. [12] and Murakami *et al*. [13], is also more indicative of slow-growing schwannomas.

Definitive diagnosis relies on histopathology and immunohistochemistry. Microscopy revealed the classic biphasic pattern of benign schwannoma with Antoni A and B areas, nuclear palisading, and Verocay bodies, a pattern foundational to diagnosis [4–7, 9, 10]. Immunohistochemistry is crucial for differentiation. The tumor cells showed strong, diffuse S-100 protein positivity [4–7, 9] and were negative for Epithelial Membrane Antigen (EMA), which is typically positive in meningiomas [5–7, 9]. This S100-positive/EMA-negative profile is definitive for schwannoma. Additional markers like SOX-10 [4] or CD57 [5, 9] can further distinguish OGS from other rare tumors.

The patient presented with a two-month history of headaches, blurred vision, and papilledema. While anosmia is considered a key symptom, our review found diverse presentations (Table 1). Many patients have olfactory dysfunction [14–16], but a significant number, including ours and others [10, 17], have preserved smell. This suggests symptoms often arise from mass effect on adjacent structures like the optic apparatus and frontal lobes, causing visual issues, headaches, and seizures, rather than direct olfactory nerve involvement [7, 10, 18]. Table 2 summarizes key features from the literature.

**Table 1:** Patient demographics, clinical presentation, and olfactory outcomes.

**Study ID**	**Age**	**Sex**	**Main symptom**	**Pre-op olfaction**	**Post-op olfaction**	**Follow-up (Recurrence)**
Sousa 2025	16	M	Headaches, hyposmia, convulsion	Hyposmia	**Restored**	2 years (No)
Vp 2022	32	M	Progressive headaches	Anosmia absent	Asymptomatic	2 years (No)
Masuda 2020	13	F	Headache	Normal	NA	5 years (No)
Taha 2018	56	M	Incidental (RTA), anosmia	Anosmia	No improvement	NA (No)
Liby 2016	13	F	Seizures, papilloedema, vision impairment	Diminished sense	Residual anosmia	6 months (No)
Manto 2016	39	F	Headache, loss of smell	Anosmia (right)	NA	4 months (No)
Bohoun 2016–1	26	M	Incidental finding	Anosmia (right)	Unchanged	NA (No)
Bohoun 2016–2	24	F	Syncope	Anosmia (right)	Unchanged	NA (No)
Quick 2015–1	64	F	Headache	Anosmia (left)	NA	NA (No)
Quick 2015–2	45	F	Incidental finding, hyposmia	Hyposmia	NA	NA (No)
Kim 2015	49	F	Headache, nausea, vomiting	Preserved	**Preserved**	19 months (No)
Choi 2009	39	F	Anosmia, frontal headache	Anosmia	**Improved**	NA
Mirone 2009	38	M	Headache, vomiting, visual impairment	Slight hyposmia	NA	18 months (No)
Figueiredo 2009	49	M	Headache, loss of smell	Bilateral anosmia	NA	NA
Adachi 2007	49	F	Generalized seizures	Normal	NA	2 years (No)
Murakami 2004	30	M	Intermittent headache	Normal	NA	NA (No)
Shenoy 2004	55	M	Generalized seizures	Preserved	NA	NA (No)
Praharaj 1999	45	M	Headaches, generalized seizures	NA	NA	NA (No)
Ulrich 1978	19	M	Epileptic seizures, visual loss	Unilateral anosmia	NA	3 years (No)

**Table 2:** Surgical, radiological, and histopathological characteristics.

**Study ID**	**Tumor location**	**Key imaging features**	**Surgical approach**	**Extent of resection**	**Olfactory nerve intraoperative finding**	**Final diagnosis (Confirmed by IHC)**
Sousa 2025	Right frontobasal	Cystic & solid; intense enhancement	Transcortical	Complete	NA	Schwannoma
Vp 2022	Right frontobasal	Homogeneous enhancement; peripheral edema (T2)	Minipterional	Radical	Right tract isolated	Olfactory groove schwannoma
Masuda 2020	Anterior skull base	T1 low, T2 high; strong enhancement	Interhemispheric	Gross Total	NA	Olfactory groove schwannoma
Taha 2018	Right frontal (intra-axial)	MRI not performed	Transcortical	Complete	Nerves not identified	Schwannoma
Liby 2016	Midline anterior fossa	Heterogeneous (solid-cystic)	Frontolateral	Total	Right nerve/bulb absent	Olfactory groove schwannoma
Manto 2016	Right anterior fossa	T1 hypointense, T2 hyperintense	Bilateral frontal	Total	Right tract not identifiable; left intact	Typical Schwannoma
Bohoun 2016–1	Right anterior fossa	T1 hypointense, T2 hyperintense	Frontobasal	Total	Continuity with right bulb	Schwannoma-like tumor
Bohoun 2016–2	Right subfrontal	T1 hypointense, T2 hyperintense	Frontal	Gross Total	Arose from right bulb	Schwannoma-like tumor
Quick 2015–1	Frontal base, nasal invasion	Solid mass with cystic areas	Bifrontal	Gross Total	Left nerve destroyed	Schwannoma WHO I
Quick 2015–2	Olfactory groove	Contrast-enhancing	Fronto-lateral	Gross Total	Left nerve destroyed	Schwannoma WHO I
Kim 2015	Left frontal base	T1 hypointense, T2 cystic; variegating enhancement	Subfrontal	Complete	**Nerve preserved**	Olfactory groove schwannoma
Choi 2009	Ethmoid to right frontal	Inhomogeneous; solid & rim enhancement	Bifrontal, subfrontal	Total	Olfactory bulb involved	Schwannoma
Mirone 2009	Olfactory groove	Cystic mass with edema; strong enhancement	Frontal, subfrontal	Complete	Left bulb/tract not identifiable; right preserved	Typical Schwannoma
Figueiredo 2009	Olfactory groove	T1 isointense, T2 hyperintense	NA	Complete	Both tracts involved	Schwannoma
Adachi 2007	Olfactory groove	T1 low, T2 high; partial enhancement	Left frontal	Total	Nerve stretched thin	Schwannoma
Murakami 2004	Left anterior skull base	Hypovascular (angiography)	Left frontal	Total	Left bulb involved; proximal tract intact	Schwannoma
Shenoy 2004	Midline anterior fossa	T1 hypointense, T2 hyperintense; honeycomb enh.	Frontotemporal	Total	Ipsilateral bulb thinned; contralateral preserved	Schwannoma
Praharaj 1999	Right frontobasal	Uniformly enhancing (CT)	Bifrontal	Total	Bulbs not identified	Hyalinised Schwannoma
Ulrich 1978	Right anterior fossa	NA	Frontal	Total	Right bulb likely destroyed	Schwannoma

The patient’s uneventful postoperative recovery and planned monitoring align with the generally excellent prognosis of OGS after gross total resection. The literature shows minimal recurrence rates, with no recurrences in reviewed cases despite follow-ups ranging from months to five years [4, 5, 10, 17, 19]. The straightforward surgical access and gross total resection achieved in this case, without the need for complex reconstruction, further support the favorable management outcome for these lesions.

## Conclusion

The diagnostic trajectory of this patient highlights the necessity of clinical vigilance in the evaluation of anterior cranial fossa masses. The radiological findings indicated a meningioma; however, the definitive diagnosis of an olfactory groove schwannoma was confirmed through detailed histopathological examination, which revealed classic schwannoma architecture supported by a definitive S100-positive and EMA-negative immunohistochemical profile. This case highlights the significance of olfactory groove schwannomas, which, despite their rarity, must be considered in diagnostics, as precise identification is essential for informing surgical management. This case demonstrates that gross total resection provides an excellent prognosis, with the possibility of definitive cure and long-term disease control.
